# Comparison of intracellular and secretion-based strategies for production of human α-galactosidase A in the filamentous fungus *Trichoderma reesei*

**DOI:** 10.1186/s12896-014-0091-y

**Published:** 2014-10-27

**Authors:** Wesley Smith, Jussi Jäntti, Merja Oja, Markku Saloheimo

**Affiliations:** VTT Technical Research Centre of Finland, Espoo, FI-02044 VTT Finland

**Keywords:** Therapeutic protein, Human α-galactosidase A, *Trichoderma reesei*, Protein body

## Abstract

**Background:**

*Trichoderma reesei* is known as a good producer of industrial proteins but has hitherto been less successful in the production of therapeutic proteins. In order to elucidate the bottlenecks of heterologous protein production, human α-galactosidase A (GLA) was chosen as a model therapeutic protein. Fusion partners were designed to compare the effects of secretion using a cellobiohydrolase I (CBHI) carrier and intracellular production using a gamma zein peptide from maize (ZERA) which accumulates inside the endoplasmic reticulum (ER). The two strategies were compared on the basis of expression levels, purification performance, enzymatic activity, bioreactor cultivations, and transcriptional profiling.

**Results:**

Constructs were cloned into the *cbh1* locus of the *T. reesei* strain Rut-C30. The secretion and intracellular strains produced 20 mg/l and 636 mg/l of GLA respectively. Purifications of secreted product were accomplished using Step-Tactin affinity columns and for intracellular product, a method was developed for gravity-based density separation and protein body solubilisation. The secreted protein had similar specific activity to that of the commercially available mammalian form. The intracellular version had 5-10-fold lower activity due to the enzymes incompatibility with alkaline pH. The secretion strain achieved 10% lower total biomass than either the parental or the intracellular strain. The patterns of gene induction for intracellular and parental strains were similar, whereas the secretion strain had a broader spectrum of gene expression level changes. Identification of the genes involved indicated strong secretion stress in the secretion strain and to a lesser extent also in intracellular production. Genes involved in the unfolded protein response (UPR) and ER-associated degradation were induced by GLA production, including; *hac1*, *pdi1*, *prp1*, *cnx1*, *der1*, and *bap31*.

**Conclusions:**

Active human α-galactosidase could most effectively be produced intracellularly in *Trichoderma reesei* at >0.5 g/l by avoidance of the extracellular environment, although purification was challenging due to specific activity losses. Strain analysis revealed that in addition to the issues with secreted proteases, the processes of secretion stress including UPR and ER degradation remain as bottlenecks for heterologous protein production. Genetic engineering to eliminate these bottlenecks is the logical path towards establishing a strain capable of producing sensitive heterologous proteins.

**Electronic supplementary material:**

The online version of this article (doi:10.1186/s12896-014-0091-y) contains supplementary material, which is available to authorized users.

## Background

*Trichoderma reesei* is a filamentous fungus known primarily for its use as a large scale producer of industrial proteins [1]. It is established within industry particularly for its ability to secrete high yields of active enzymes. This potential is an attractive benchmark to follow when considering the production of more sensitive heterologous proteins of significant value and therapeutic application, particularly in light of its compatible basis for humanised glycosylation engineering [2] and GRAS status of *T. reesei*. A limited number of mammalian proteins have been produced successfully in this system, including bovine chymosin [3] and antibody Fab fragments [4]. One salient feature of the host is its aggressive and opportunistic nature regarding nutrition uptake [5], with a broad use of complex carbon sources. Such opportunism often causes issues with the stability of heterologous proteins [6]; whereby the harsh extracellular proteolytic environment [7] directly degrades the sensitive recombinant products. Comparison with other potential systems for microbial production of high value proteins demonstrates not only the potential but also some clear limitations of *T. reesei* [8]. Optimisation of the production strategy is therefore one avenue that may be explored to bypass and also to define these limitations, leading to improved yields of high-value target proteins.

The most commonly used technique to enhance heterologous protein production in *T. reesei* is by fusion of the target protein with a secretion carrier, generally the catalytic domain and linker of the major cellulase enzyme cellobiohydrolase I. Carrier fusions of this type have been proposed to have a diversely positive effect on production levels by stabilising mRNA, improving intracellular trafficking in the secretory pathway, and proximity shielding against proteolysis [9]. An alternative strategy can be to direct intracellular accumulation of a target protein, previously highlighted by the hydrophobin fusion strategy leading to protein body (PB) formation [10,11]. Another self-assembling protein that can promote intracellular accumulation and PB formation is derived from the maize gamma zein storage protein [12], ZERA®. This fusion technique relies on ZERA’s natural ability to accumulate in the ER and on ZERA-ZERA interactions driven by intermolecular disulphide bonds, recruiting the individual fusion proteins into PB structures. Protein body fusions of this nature have a tight conformation and are surrounded by the ER membrane, thereby insulating the foreign protein from other cell components and avoiding proteolysis to some extent during cell disruption and PB harvesting.

Human alpha galactosidase A (GLA) was chosen as a high value model protein for this study on the basis of its current use in the treatment of Fabry disease [13] by enzyme replacement therapy. GLA is currently produced commercially in Chinese Hamster Ovary (CHO) cells [14]. Fabry disease itself is an X-chromosome-linked lysosomal storage disease caused by loss of native GLA activity in affected individuals, allowing a toxic build-up of the substrate globotriaosylceramide. GLA in its mature form is a 46 kDa glycoprotein with four N-glycosylation sites and five disulphide bridges, and it is active as a 102 kDa homodimer. Enzymatic activity is thought to be independent of the glycosylation pattern, which is more likely to be important for structural integrity, solubility, targeting, and antigenic compatibility [15].

The aim of this study was primarily to investigate whether authentic and functional GLA can be produced in *T. reesei,* and to compare intra- and extracellular production strategies in an attempt to reach industrially significant production levels of active enzyme. A secondary aim following this comparison was to reveal the physiological consequences of the two production strategies. Transcriptional profiling and other techniques were used to elucidate the cellular mechanisms causing bottlenecks in production. Equivalence of a fungal version of human GLA in terms of yield and enzyme functionality would be a clear indication of the potential of *T. reesei* as an economically valid microbial system for the production of human therapeutic proteins.

## Methods

### Cloning and strain construction

**Figure 1:**
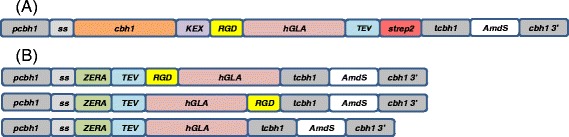
**Map of DNA constructs. (A)** The construct was designed to secrete the target protein human GLA (*hGLA*) under the *cbh1* promoter (*pcbh1*) with the aid of a CBHI secretion signal (*ss*) and CBHI carrier fusion (*cbh1*), which was to be proteolytically cleaved at a KEX2 site (*KEX*) on route to secretion. A TEV protease site (*TEV*) was also included for downstream polishing after purification by Strep Tactin affinity using a strep2 tag (*strep2*) **(B)** Three variations of GLA were designed with RGD integrin (*RGD*) targeting for intracellular accumulation by ER retention of the Zera® peptide system (*ZERA*). The *cbh1* locus was used as an integration site for all the constructs by homologous recombination using the *cbh1* promoter sequence and a section of DNA downstream of the *cbh1* region (*cbh1 3′*). The native terminator sequence (*tcbh1*) was followed by an *Aspergillus nidulans AmdS* (*AmdS*) marker gene for selective growth on acetamide. The size of the protein expressing region of the secretion construct was 2775 bp and 1632 bp for both RGD containing intracellular constructs whereas the control construct lacking RGD was 1563 bp.

Constructs for intracellular GLA production were made from a synthetic gene coding for ZERA-GLA [12], obtained from Geneart (Regensburg, Germany). GLA [NCBI Gene ID: 2717] was optimised to *the T. reesei* genomic codon bias and 40 bp homologous overlaps with the destination vector pHH01 [16] were included to excise a *Pac*I site upstream and downstream respectively; CATCTTTTGAGGCACAGAAACCCAATAGTCAACCGCGGAC, GACCTACCCAGTCTCACTACGGCCAGTGCGGCGGTATTGG. pHH01 is a *T. reesei* expression vector with a yeast backbone and sequences for amplification in *E. coli,* also containing an acetamidase selection marker, and a *cbh1* promoter and terminator with 3′ integration flanks to the *cbh1* locus in *T. reesei* (Figure 1). The secreted GLA construct was made by nested PCR cloning using Phusion HF polymerase (Thermo Fisher Scientific Inc. Waltham, MA, United States of America) according to the manufacturer’s two step protocol conditions with a *cbh1* DNA template and the synthetic GLA plasmid. A forward oligo was used, overlapping pHH01 followed by a *cbh1* priming region (CATCTTTTGAGGCACAGAAACCCAATAGTCAACCGCGGACATCATGTATCGGAAGTTGGCCGTCATCTCGG) with a reverse oligo designed to introduce a KEX2 protease site between the native CBHI carrier and recombinant GLA (GCGCTGGCGGTGTAGGTGGTGCGCTTGTCCATGGGAGGTCCGGGAGAGCTTCCAGTG). After three polymerase chain reaction cycles an additional reverse oligo was added to the mix containing a GLA priming region with an extra terminal sequence to code for a strep2 tag with TEV protease site. (CCAATACCGCCGCACTGGCCTCACTTCTCGAACTGGGGGTGGCTCCAGCCCTGGAAGTAGAGGTTCTCGCCGCCACCGCCGAGGAGGTCCTTGAGGCTCA). A further 30 cycles were performed in order to anneal the entire nested product which contained 20 bp overlaps for each piece. Yeast homologous recombination cloning was used for all of the constructs. The expression vector pHH01 was linearised with a *Pac*I (New England Biolabs Inc. Ipswich, MA, USA) restriction enzyme between the *cbh1* promoter and terminator and transformed into *S. cervisiae* together with the ZERA-GLA and CBHI-GLA cloning fragments designed to replace the *Pac*I site and recircularise the plasmid. The transformation mixes were plated on SCD-Ura [17] for URA3 strain selection and the recombinant yeast plasmid DNA was then isolated and transformed to the *E. coli* DH5 alpha. Positive clones were identified by restriction enzyme digestions and DNA sequencing. The *T. reesei* expression cassettes shown in Figure 1 were excised from the cloning vectors using dual *PmeI* sites (Thermo) and transformed into *T. reesei.* Transformations were made using the Rut-C30 strain including a mus 53∆ [18] mutation to enhance genome-targeted integration according to the standard protoplast-based protocol [19]. Genomic Integration into the *cbh1* locus was confirmed by PCR screening from transformants successfully growing on Trichoderma Minimal Medium (TrMM) acetamide selection plates [19] after two successive streaks made on selective medium. Uninuclear clones were obtained by plating single colonies from spore suspensions onto selection plates and finally amplified for spore culturing stocks on potato dextrose DIFCO 213400 agar plates (Becton, Dickinson and Co. New Jersey, USA).

### Culturing

Small scale 300 ml cultivations were performed in two litre shake flasks with TrMM buffered to pH 4.8 with 100 mM piperazine-n, n’-bis 3-propanesulfonic acid, and supplemented with 4% lactose Fagron 208090–0002 (Fagron Nordic A/S. Copenhagen, Denmark), 2% soluble spent grain (extracted from solid grain material (Harbro Ltd. Turriff, Aberdeenshire, United Kingdom) at pH 5.0 by adjustment with NaOH and using a 115°C heating step for 10 mins before centrifugation to obtain the soluble fraction), and 0.2% peptone. Five separate transformants were grown for each construct and compared for growth and expression levels in order to establish a representative clone for upscale culturing. Protease inhibition cultivations for the CBHI-GLA secretion strain were performed in Whatman™7701-5102 24 well culture plates (GE Healthcare UK Ltd. Little Chalfont, Buckinghamshire, United Kingdom) with the same medium in 4 ml volume including daily dosing of 10 nM pepstain A SIGMA P5318 (Sigma-Aldrich Co. LLC. St. Louis, MO, United States of America), 10 nM chymostatin SIGMA C7268, 5 mM EDTA, and 0.2 mg/ml trypsin inhibitor SIGMA T9128.

Larger scale bioreactor cultivations were performed in order to obtain sufficient material for purification of comparable test batches. 20 litre cultivations in a Braun Biostat C-DCU3 bioreactor (Sartorius AG. Göttingen, Germany) were started with a 10% inoculum, grown on 40 g/l lactose with 33% (v/v) of a soluble extract of spent grain. The bioreactor medium contained 60 g/l lactose with 40% (v/v) of the same spent grain extract. Cultivation conditions were controlled as follows; pH was maintained at 4.5 ± 0.5 using NH_4_OH, foaming was controlled by automatic addition of Dow Corning 1500 antifoam agent when required (Dow Corning Corp. Midland, Michigan, United States of America), dissolved oxygen was maintained above 30% by agitation (400–550 rpm) and oxygen enrichment of the gas flow (0-66%) at a constant gas flow rate of 8 l/min, and temperature was controlled at 28°C. A fed-batch cultivation with lactose feeding (200 g/l) starting at the end of the batch phase was controlled by an algorithm based on the consumption of base for pH control [20,21].

Bioreactor cultivations for transcriptional profiling were performed in triplicate from a single inoculum per strain in one litre Sartorius Q DCU4 bioreactors using 4% lactose and 2% spent grain extract; all other parameters were controlled as in the previous batch method.

### Protein gels and Western blot analysis

SDS PAGE samples were made with LI-COR 928–40004 protein sample loading buffer (LI-COR Biosciences Inc. Lincoln, NE, United States of America), a pre-cast Biorad Criterion gel system (Bio-Rad Laboratories Inc. Hercules, CA, United States of America) and blotted by a semi dry Biorad Transblot Turbo Western blotting system. Blots were then probed with primary antibody (anti ZERA rabbit αR8 1:7000 [12], Santacruz anti GLA H104 rabbit 25823 1:1000 (Santa Cruz Biotechnology Inc. Dallas, Texas, United States of America), iBA StrepMAB classic 21507001 1:2000 (IBA GmbH. Göttingen, Germany), anti CBHI MAB261 1:1000 [22]) and quantified using a fluorescent secondary antibody: ODYSSEY IR Dye 680RD 92668071 1:10000/IRDye 800CW 92632710 1:10000 (LI-COR) and a LI-COR ODYSSEY CLX system for near infrared scanning and quantification). Quantifications were based on a moving average curve fit from five different dilutions of a protein standard. A representative batch of ZERA-GLA was chosen as a positive control and quantification standard by first quantifying against a commercial GLA from *E. coli* 12078-H08H (Sino Biological Inc. Beijing, China) using GelCode Blue G250 Coomassie stain (Thermo). Duplicate protein samples were used for each quantification. The densitometry calculations were processed with ImageQuant TL software (GE Healthcare) based on a best fit curve of five GLA standard dilutions. CBHI carrier quantities within the culture sample were determined against a pre-quantified batch of CBHI control protein (VTT Technical Research Centre of Finland, Espoo, Finland).

### Purification of GLA

**Figure 2:**
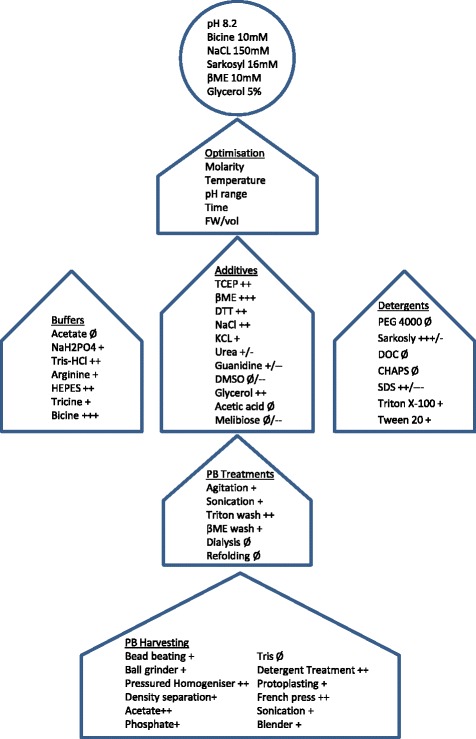
**Schematic workflow of the protein purification protocol development.** Biomass for the intracellular production strains was extracted by various PB harvesting techniques and eventually solubilised under many different conditions requiring stepwise optimisation. The effects at each step were measured by Western blotting for yield increase and by activity assay, to determine whether integrity of the GLA enzyme fusion was retained. The harvesting, PB treatment and solubilisation methods tested are shown in the arrows, and the final solubilisation conditions are shown in the circle on the top. A plus sign (+) represents an increase in active yield and two or three such signs show the approximate magnitude of this effect. Similarly one to three negative signs (−) denote the magnitude of active yield decrease. A null effect sign (Ø) was used where there was no apparent change in active yield. Some additives have both signs to show that the effect of active yield was dictated by a change in molarity of the additive.

Mycelial biomass from ten litres of fermentation broth was harvested by vacuum filtration on double-layered 240 mm diameter Whatman GF/A filter paper (GE Healthcare) in a Büchner apparatus and washed with reverse osmosis water before being frozen at −20°C for storage. Many different buffer conditions and methods for cell breakage, PB extraction and solubilisation were tested both for protein and enzyme activity yield, as briefly outlined in Figure 2, before a standard protocol was determined. In this protocol 1 g of the cell material was resuspended in 10 ml of 10% sucrose, 10 mM NaH_2_PO_4_ ( pH6.5)_,_ 1% Triton x100, 2 mM EDTA, 1 mM Phenylmethanesulfonyl fluoride Sigma P7626 and 10 μM Pepstatin A and homogenised by a 10 mm Polytron PT1200 disrupter probe (Kinematica AG. Luzern, Switzerland). The homogenous material was then subjected to three passes through an SLM Aminco FA030 French press (Horbia Instruments Inc. Irvine, CA, United States of America) at 20,000 psi. Initial separation of PBs from leftover biomass was performed in 50 ml tubes by centrifugation for 5 minutes at 200 × g. After separation from the mycelial pellet, the supernatant containing soluble cell extracts, small aggregates and the protein bodies was then used to enrich the protein bodies by centrifugation at 2000 × g for 10 minutes. Two washes were then performed on the pellet material using 10 mM NaH_2_PO_4_ (pH6.5) and 1% Triton-X100, to remove any endoplasmic reticulum membranes. PB-enriched aliquots were then made from one millilitre of original material and frozen for solubilisation method development.

The solubilisations were based on a controlled concentration ratio from disrupted biomass, e.g. 1 g of mycelial fresh weight was disrupted in 10 ml of breakage buffer, and PBs were then enriched into a small pellet and reconstituted in 1 ml of solubilisation buffer to a maximum concentration of approximately 2.5 mg/ml ZERA-GLA. A buffer containing 50 mM Bicine pH 8.2, 5% Glycerol, 150 mM NaCl, 16 mM Sarkosyl, 10 mM β-mercaptoethanol was used to resuspend the PB pellet before treatment in a Fritsch Laborette 17.202 sonic bath (Fritsch GmbH. Idar-Oberstein, Germany) for one minute. The suspended PB pellets were left to solubilise overnight at +8°C on a Brunswick TC7 roller drum (Eppendorff AG. Hamburg, Germany). The soluble fraction was recovered by centrifugation at 20,000 × g for 30 minutes. Proteolytic cleavage of the GLA enzyme from ZERA was achieved by use of a monomeric TEV protease Invitrogen AcTEV 12575 according to the manufacturer’s instructions (Thermo).

Secreted GLA was purified using an IBA 2-1531-001 Strep-Tactin 1 ml flow column by adjustment of the sample pH to 7.5 using NaOH and elution by acid-driven disassociation in 50 mM sodium acetate activity buffer, pH 4.5 according to the manufacturer’s instructions.

### GLA activity assay

Activity measurements were made by mixing a 25 μl enzyme sample into 100 μl of the 2.46 mM fluorescing substrate 4-methyl-umbelliferyl-alpha-D-galactopyranoside Sigma M7633 in 10 mM acetic acid, pH 4.5. The enzyme reactions were continued for one hour on an orbital shaker at 37°C before being stopped by addition of 1.25 ml 200 mM glycine solution at pH 10.4. A standard curve was prepared of 0–500 ng/ml of 4-methylunbelliferone Sigma M1381. All samples were analysed at an excitation wavelength of 365 nM and read at 450 nM on a Varioskan (Thermo). Activity units were expressed as μmol of 4-methylumbelliferone/h/mg protein.

### RNA isolation and array hybridisation

Mycelial samples from the one litre bioreactors were collected by vacuum filtration on Whatman GF/A filter paper and then rinsed in 0.9% NaCl and frozen immediately in liquid nitrogen. RNA was isolated from 50 mg of the mycelia with RNeasy Plant RNA isolation kit (Qiagen GmbH. Hilden, Germany). RNA quality was assessed for purity and quantity using an Agilent RNA 6000 analyser (Agilent Technologies Inc. Santa Clara, CA, United States of America) The purified RNA was then converted into cDNA using an Invitrogen Superscript Double-Stranded cDNA Synthesis Kit (Thermo) prior to labelling with Cy3 One-Colour fluorescent dye (Roche NimbleGen Inc. Basel, Switzerland) according to the manufacturer’s instructions. The labelled cDNA was hybridised on an automated HS12 system (Roche) to custom-made four plex microarray slides (Roche) designed from the *T. reesei* genome version 2.0 [23,24]. Analysis was performed in an MS200 microarray scanner (Roche) to identify signal probe intensity of the six 50-74mer probe replicates designed from the known and predicted genes in *T. reesei*.

### Microarray data analysis

R/Bioconductor (http://www.r-project.org) was used for all data analysis. The raw data was normalized with Robust Multichip Average (RMA) normalization [25]. The quality of the microarray data was assessed on the basis of a report from the arrayQualityMetrics–package [26] and by observation of the distribution of the log_2_ intensities on each array before and after normalization (Additional file 1A). Additionally, the similarity of replicate samples to each other was verified by plotting the arrays on a two dimensional display using principal component analysis (Additional file 1B).

Statistical differences in expression were analysed using linear modelling with tools of the limma package [27]. For each gene, a linear model was fitted by the least squares method and differential expression within pairs of experimental conditions was computed using an empirical Bayesian approach [28]. Genes with log_2_-scale fold change > +\-1 were considered to be up- or down-regulated, respectively. The significance test results were visualized as volcano plots showing the expression level difference (log_2_-transformed fold change) versus the significance of the change (−log_10_ of the p-value). The volcano plot shows, at a glance, the number of changes between the strains being compared. In a large and outspread volcano plot, genes in the top-left and top-right corners of the plot are most significantly down- or up-regulated. A small and shallow volcano indicates that there were no significant changes between the conditions, i.e. that the changes in the expression levels were small (below twofold) and insignificant (p-value >0.001).

## Results

In order to compare the two different production strategies of human α-galactosidase in *T. reesei*, four expression constructs were made from a synthetic gene codon-optimised to *T. reesei* codon usage and transformed into the fungus (Figure 1). In one construct GLA was fused to the *T. reesei cbh1* catalytic domain and linker region in order to provide a secretion carrier. For the remaining constructs GLA was fused with ZERA peptide to promote protein body formation inside the cell. Three of the constructs had RGD peptides attached at either end of GLA to enhance its localisation in the lysosome for its use in enzyme replacement therapy for humans. The transformants obtained for each of these constructs were purified through single spore cultures, and integration of the expression construct to the *cbh1* locus was verified by PCR analysis.

### Production of human α-galactosidase in *T. reesei*

**Figure 3:**
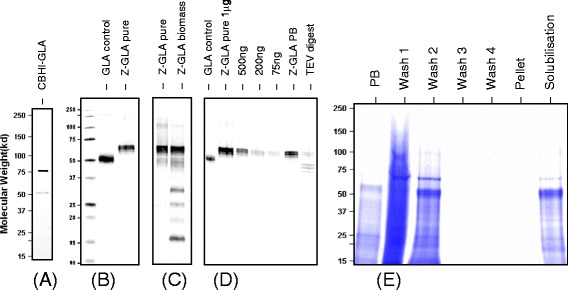
**SDS-PAGE and Western blot analysis of the proteins produced. (A)** Secreted CBHI-GLA purified via strep2 affinity, measured by strep-tactin-AP Western blot (100 ng loaded). The 50 kD band represents the GLA cleaved by KEX2 protease from the CBHI carrier. The larger band remains as a CBHI-GLA fusion. **(B)** Comparison of a solubilised batch of purified ZERA-GLA (Z-GLA pure) compared to commercial GLA (GLA control) (1 μg of each). The ZERA-GLA protein runs about 10 kD higher than commercial GLA as expected. **(C)** The same ZERA-GLA batch compared to ZERA-GLA contained in mycelium (Z-GLA Biomass) and extracted directly from filtered mycelia by boiling in protein sample loading buffer (1 μl of each). Detection by anti ZERA Western blot reveals the presence of several lower molecular weight degradation products contained within the cell, still retaining the ZERA peptide. **(D)** A TEV protease cleavage of GLA from the ZERA peptide (TEV digest) directly using a 10-fold digest buffer dilution of insoluble enriched protein body material (Z-GLA PB), compared to 0.5 μg of commercial GLA and 1 μg of the purified soluble form of ZERA-GLA. 5 μl were loaded for both the digest and starting PB material. The anti GLA Western blot shows that while cleavage is possible from insoluble PBs three lower molecular weight forms of GLA are present, perhaps due to endogenous protease. **(E)** A four step pellet wash purification of the ZERA-GLA PB material from a 5 μl sample of 1 g fresh weight diluted in 10 ml (PB) showing the soluble components removed from the PB material with a 5 μl sample of tenfold concentration increase (Wash). The PB pellet is then solubilised in the same volume and centrifuged leaving insoluble debris (Pellet) and the soluble ZERA-GLA (Solubilisation) (5 μl for both).

Five individual transformants for each of the Intracellular ZERA-GLA variants and CBHI-GLA secretion strain were grown in replicate shake flask cultures. GLA production yields quantified by near infra-red Western blots were approximately 90 mg/l for all the intracellular ZERA-GLA variants, whereas the secreted CBHI-GLA product was barely above the limit of detection. No growth defects were observed and there was no significant variation in production levels between the replicates, and therefore one representative clone for each strain was chosen for up-scaled production. Secreted CBHI-GLA was deemed unsuitable for bioreactor studies due to its instability and low GLA yield. However, its production level was eventually improved to around 20 mg/l in 24-well plate cultures and to a total of 60 mg/l including CBHI carrier bound forms by addition of a cocktail of protease inhibitors (Figure 3A). The strain expressing ZERA-GLA with C-terminally attached RGD peptide was grown in a 20 litre batch cultivation and ZERA-GLA lacking RGD peptide was produced in a 20 litre fed batch cultivation based on the monitoring observations of the previous batch run.

Batch cultivation for ZERA-GLA produced 267 ± 18 mg/l and the subsequent fed-batch bioreactor run with extended lactose feeding increased the yield to 636 ± 41 mg/l. When including proteolytic cleavage products in the quantification, the total detected amount found in the cells was around 1200 mg/L (Figures 3B and C). In both the batch and fed-batch cultivations growth was similar to that of the parental strain and peak expression of ZERA-GLA was achieved at the end of the cultivations when CO_2_ levels had decreased almost to the baseline after exhaustion of carbon source.

### Purification and enzyme activity measurements of human GLA

**Figure 4:**
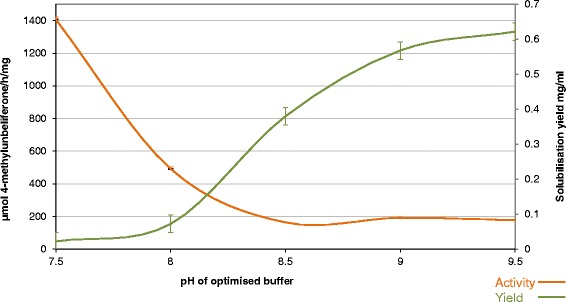
**Enzyme activity in relation to recovered yield from insoluble material.** A pre-pellet washed batch of PB material was solubilised over a neutral to alkaline pH range and tested for target protein concentration by quantitative Coomassie stain and GLA enzyme activity measurement. A specific activity loss trade-off vs. concentration yield, by solubilisation pH change was established to determine the optimum pH for the procedure.

In the ZERA peptide protein body purification concept the target protein is purified from lysed cell material based on the high density of the PBs. Developing a protocol for GLA isolation and solubilisation required extensive trials and optimisation (Figure 2), but ultimately a relatively robust protocol was established. In our initial purification trials we focused on optimising recovery of PB material from the mycelia. In the case of mild disruption methods such as the use of homogenisation blenders, PBs could not be extracted efficiently. In more harsh disruption methods such as grinding and bead beating, the PBs were also disrupted and consequently could not be recovered by density separation. Disruption in a French press with added Triton detergent allowed for sufficient disruption of the mycelial biomass without harming the PBs, which could subsequently be harvested efficiently by differential gravity separation. The remaining challenge of PB solubilisation was approached by testing a number of additives and detergents in a range of different buffers (Figure 2). These were then individually assessed for solubilisation yield and GLA activity and the best conditions were combined and optimised. During this method development phase for PB solubilisation it became apparent that loss of specific activity was a major challenge. Highly concentrated batches of >2 mg/ml of ZERA-GLA could be made in 50 mM Bicine buffer, pH 9.5, with the addition of 50 mM ionic surfactants, either SDS or Sarkosyl , 900 mM NaCl and 50 mM BME for reduction of the ZERA intramolecular disulphide bridges. This led to a clear protein enrichment of ZERA-GLA from cell lysate material (Figures 3D and E) with greater than 60% total recovery of material and >70% purity as determined by Coomassie staining. Unfortunately these conditions led to a major reduction of enzyme activity, with eventual total loss after one freeze thaw cycle. Optimisation of the pH and molarities of the solubilisation buffer components led to retention of a small proportion of the enzyme activity by trade-off with solubilisation yield (Figure 4). After optimisation, product concentrations of 0.1-0.2 mg/ml were observed with a 5-10-fold lower specific activity than the commercially available CHO-derived enzyme. Proteolytic release of GLA enzyme from the ZERA fusion could then be achieved at the TEV cleavage site using a monomeric TEV protease (Figure 3D); the specific activity after this polishing procedure remained unchanged.

The specific activity of CBHI carrier-secreted GLA collected by Strep tag purification was comparable to that of the commercial enzyme Replagal (results not shown). However, this method of production and purification was unreliable due to high levels of product degradation and loss. Secreted GLA became unstable during the Step-Tactin binding process when the acidic culture pH was increased to the neutral pH range of Strep-Tactin binding conditions, despite the addition of protease inhibitors.

### Physiological consequences of the production strategies

**Figure 5:**
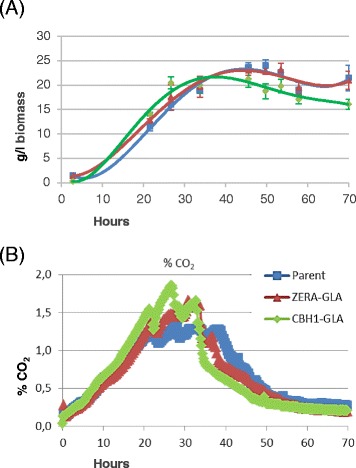
**Bioreactor data.** Batch bioreactor cultivations in triplicate. The three production strains were cultured in triplicate for the parental strain (Parental), intracellular production strain (ZERA-GLA) and secretion strain (CBHI-GLA) and then analysed by transcriptional profiling for physiological observations. **(A)** Cell dry weight determined over time in grams of dried biomass per litre of culture volume. **(B)** Carbon dioxide monitoring was determined over time and measured in percent dissolved CO_2._

In order to establish the specific physiological and transcriptional changes as a result of either secreted or intracellular production, bioreactor cultivations were performed in triplicate for each of the parental, GLA-secreting and intracellular production strains. Quantification of cell dry weights and CO_2_ formation from the cultivations revealed that growth of the GLA-secreting strain peaked about 15 hours earlier than both the parental and intracellular ZERA-GLA strains (Figure 5). The secretion strain also produced about 10% lower peak biomass. However, according to both dry weight and CO_2_ measurements the maximal growth rates of the three strains were rather similar.

**Figure 6:**
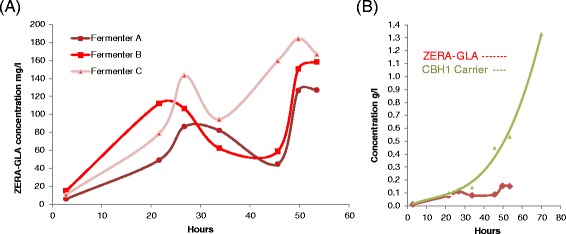
**Total yield of target protein production during the bioreactor cultivations. (A)** Triplicate cultivations of ZERA-GLA intracellular production showing total ZERA-GLA expressed over time by quantification from anti-ZERA Western blot analysis. Two peaks of ZERA-GLA production are apparent, separated by a decline in the ZERA-GLA level at approximately 30 to 45 hours of the culture. **(B)** A comparison of CBHI carrier produced as a result of CBHI-GLA production by secretion vs. intracellular ZERA-GLA production over time. By assessing the more stable CBHI carrier by quantitative anti CBHI Western instead of the unstable secreted GLA product it is clear to note that despite the eventual loss of secreted GLA through proteolysis more total GLA was actually produced than with the intracellular ZERA-GLA (quantified by anti ZERA Western).

The accumulation of intracellular ZERA-GLA was measured for all replicates (Figure 6). The best producer was the intracellular ZERA-GLA strain, at 150 mg/l. These production levels were compared over time, revealing an unexpected pattern of accumulation with two separate production peaks. ZERA-GLA came to an initial production peak at around 100 mg/L at 24 hours before decreasing to ca. 50 mg/L at 35–40 hours and finally recovering to 150 mg/L after 48 hours. Analysis of the secretion strain showed that despite the instability of GLA in culture medium, much higher quantities of the CBHI carrier fusion partner were accumulated. Levels of proteolytically cleaved CBHI carrier around 1.3 g/l were observed, precisely tenfold lower than the 13 g/l of endogenous CBHI that was produced in the parental strain (data not shown).

**Figure 7:**
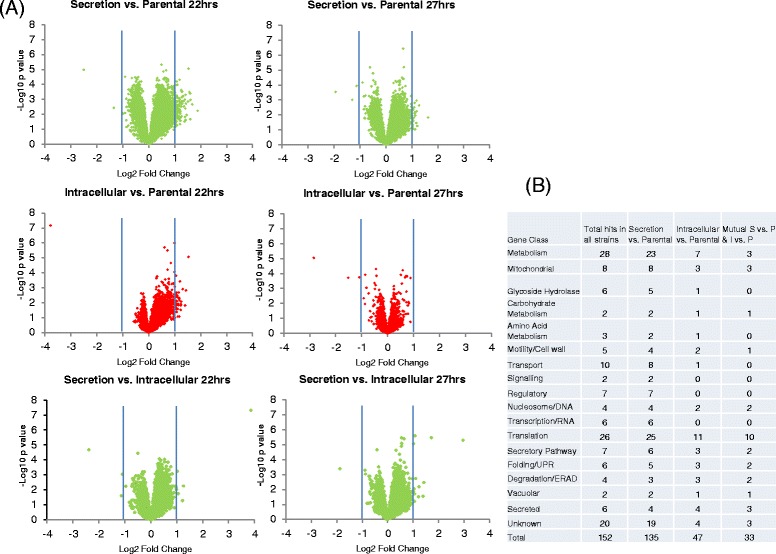
**Transcriptional profiling data comparing the production strategies. (A)** Volcano plots of the combined triplicate RNA microarray data at the points of highest interest, 22 and 27 hours showing the spread of gene expression events in terms of up and down regulation between each production strategy comparison. **(B)** A table showing the transcriptional changes exceeding the 2-fold change threshold for each comparison and also listed by gene category throughout the cultivations. The total number of hits shows the combined number of transcriptional changes for all of the production strategies and the final column shows the mutual transcriptional changes observed between secretion and intracellular production. In the case of intracellular production most of these transcriptional changes observed are also found in the mutual category showing that many similar genes are highlighted between both types of production.

Transcriptional profiles for parental, secretion and intracellular production strains were determined by quantitative comparison of mRNA levels measured by microarray. The production strategies were compared at 22 hours during the mid-logarithmic growth phase and after 27 hours at the late-logarithmic growth phase of cultivation. To identify differentially expressed genes, we performed significance tests comparing two strains at a time. The significance test results were visualized as volcano plots showing the expression level difference between the two strains (log_2_-transformed fold change) versus the significance of the change (−log_10_ of p-value). These volcano plots (Figure 7A) showed that in the secretion strategy the mRNA transcript levels of many genes had changed compared to the parental strain, in most cases by an increase in gene transcription. The gene expression during the intracellular production strategy is relatively similar to that of the parental strain, the only significant shift in data spread being at 22 hours when a small number of up-regulated genes were over twofold induced. In general the transcriptional changes observed followed a similar pattern when progressing to the 27 hour time point, albeit at a reduced level of fold change. Further analysis of the secretion vs. intracellular strain at these relevant time points, and comparison of the genes with significant changes, demonstrated that similar changes had occurred for both forms of production, although the relative level of fold change in regulation was lower for the intracellular production and often did not exceed the twofold change threshold. The listing of genes up-regulated during GLA production by category shows that genes involved primarily in metabolism, transport, and translation were enriched (Figure 7B).

Genes related to the secretion pathway are obviously of special interest. A number of genes involved in UPR and protein folding or degradation in the ER (ERAD) are induced (Additional file 2). These induced genes include *hac1*, encoding a transcription factor of UPR, the protein disulphide isomerase *pdi1*, PDI-related *prp1*, the chaperone calnexin *cnx1*, and the ERAD pathway genes *der1* and *bap31*. This observation suggests that both the secretion strain and the intracellular strain suffer from secretion stress. As the extent of induction of these genes is higher in the secretion strain, it appears that this strain suffers more strongly from secretion stress than the intracellular production strain. This can be shown specifically by analysing the differences in gene induction; for example *hac1* and *der1* are above the twofold change threshold in the secretion strain at 2.23 and 2.02, but below the threshold in the intracellular strain at 1.45- and 1.73-fold.

## Discussion

Human GLA was produced in *T.reesei* to >0.5 g/l levels with the aid of the ZERA fusion partner. However, several noteworthy limitations of this production strategy were revealed. Attempts to produce this enzyme by the classical CBHI carrier secretion method were initially unsuccessful. It was possible to obtain low mg/l yields by addition of a strong cocktail of protease inhibitors to the culture media and harvesting at an early time-point before significant proteolytic degradation occurred (data not shown). The fact that far higher quantities of CBHI carrier lacking the GLA fusion partner were observed from these cultures, coupled with the knowledge that CBHI fusion carriers have previously been successfully used for more proteolytically stable proteins such as Fab fragments [4], demonstrates the influence of the extracellular protease profile of *T.reesei* in the degradation of sensitive heterologous proteins. Traditionally it has been challenging to produce large quantities of mammalian proteins in filamentous fungi [8], and this was the driving force to investigate the possibilities of intracellular protein production using the ZERA PB storage system. It was therefore not surprising to note the relative success of intracellularly accumulated GLA, providing further evidence that it is indeed possible to express functional mammalian therapeutic proteins in *T. reesei* when degradation by secreted proteases can be avoided.

It is important to note at this point that despite the relative success of obtaining full length GLA from intracellular accumulation, there is still an intrinsic difference between secretion ability and cell storage capacity in *T. reesei.* Quantification of CBHI carrier levels observed from the bioreactor cultures revealed a near tenfold higher production level of CBHI carrier cleaved from secreted GLA than intracellular ZERA-GLA; 1300 mg/l and 150 mg/l, respectively. This shows that despite the low yield of actual GLA observed due to proteolysis, more GLA was in fact synthesized in the strain producing secreted GLA as compared with the strain producing intracellular ZERA-GLA. Furthermore, the levels of endogenous CBHI from the parental strain were a further tenfold higher at around 13 g/l (data not shown). Clearly there are not only issues with stability of GLA during secretion or after secretion but also common cellular mechanisms capable of limiting the basal expression or retention of this human enzyme.

When considering the quality of recombinant GLA enzyme, the primary concern is its ability to be purified while retaining relevant levels of specific activity. Despite the low yields of full size GLA obtained by carrier secretion a small quantity was purified, with specific activity close to that of the commercial form obtained from CHO. This was not an unexpected result considering that *T. reesei* is a known producer of high quality recombinant enzymes; however it was significantly more challenging to reproduce similar levels of specific activity from ZERA-GLA produced intracellularly. Establishing a purification method for intracellular ZERA-GLA required greater focus, having not been previously tested in any filamentous fungi. Extraction of intact PBs from the mycelial biomass required rather harsh yet well-defined disruptive forces so as not to also disrupt the PBs. Once the PBs were obtained, the methodology to enrich and wash to some extent by gravity-based separation was rather simple and robust. In order to test the enzyme in a more comparable way, solubilisation of the PBs would be required. As the results observed above demonstrate, it was possible to solubilise ZERA-GLA under several conditions but loss of specific activity occurred to some extent in every case. The most important criteria for solubilisation of the ZERA-GLA fusion were high pH with the addition of a reducing agent and ionic surfactants. Unfortunately, these factors resulted in significant loss of enzymatic activity. It was possible by adjustment of the molarity of the solubilisation buffer components, combined with optimisation of pH, to retain some of the activity, although at the cost of lower solubilisation yield. A specific activity to yield trade-off was established at around pH 8.2 although at this pH only 10-20% of the PB material was recovered, compared with almost 100% using highly basic pH. Cleavage of GLA from the ZERA fusion by TEV protease could be achieved, but enzymatic activity did not recover. These findings are probably due to the natural environment of GLA contained within the lysosome. With its low pH optimum of 4.5, the enzyme is likely to be innately less stable and less suitable for purification in basic conditions. In the comparison of these two production strategies it is relevant to note that both methods have certain advantages and also challenges. Moreover, selection of more compatible model proteins without such stringent requirements could lead to more encouraging results.

Despite the various challenges of both secreted and intracellular modes of production, it remains pertinent to investigate further the physiological and gene level regulation changes of both, in order to shed light on the cellular processes and production bottlenecks involved. As a general summary it can be concluded that production of GLA as a secreted product fused with the CBHI carrier caused a more pronounced stress effect with many more up-regulated genes, than the intracellular ZERA strategy. This correlated with the results based on cell growth, which not only peaked earlier during bioreactor cultivation but also reached a lower overall total biomass than the parental culture. By contrast, intracellular production strains grew in an almost identical manner to the parental control. Many of the genes up-regulated were identified in both strains, but the extent of the up-regulation was higher in the secretion strain. This could mean that in the secretion strain the foreign protein is detected more strongly as it travels through the whole secretion pathway. By contrast the ZERA-GLA protein is probably only present in high concentrations in the ER before being packed into protein bodies, and is therefore not detected with equal strength. It was interesting to observe that the production levels of ZERA-GLA during fermentation occurred in two distinct peaks (Figure 6B). An early peak occurred at the end of the logarithmic growth phase, dropping to around half during stationary growth, before recovering to a final and eventually higher peak at the end of the stationary phase. The second and highest peak in production appeared to occur when maximum biomass had been reached and presumably energy resources for growth were no longer in competition with target protein production. This phenomenon of a mid-point drop in total production yield suggests the existence of mechanisms capable of clearing the heterologous protein out from the ER. This conclusion is also supported by the fact that many of the significant transcriptional changes observed point towards secretion stress. It is also possible that during secretion stress further feedback controlling down-regulation of target protein biosynthesis is initiated, causing additional yield limitations [29].

## Conclusions

Enzymatically active human GLA was successfully produced in *T. reesei* at detectable levels by CBHI carrier secretion and more significantly to >0.5 gram per litre levels by intracellular accumulation with the ZERA peptide fusion strategy. It was possible to partially purify GLA from both production modes, although loss of specific activity was a challenging aspect concerning purification of the ZERA fusion product. Transcriptional profiling showed that secretion stress is induced in *T. reesei* in both of the production modes, albeit more strongly by the secretion strategy.

## Additional files

Additional file 1:
**Data quality of the microarrays.** **(A)** Density histogram of rma normalized data. The variance of log_2_ intensities of the normalised arrays intensity fall within acceptable limits for comparison. **(B)** Boxplot of rma normalized data. The appropriate similarity of replicate samples was verified using principal component analysis in this two dimensional display. Additional file 2:
**List of significant genes in all of the comparisons.** Listing of all the significantly changing genes found above the fold change threshold in all transcriptional profiling comparisons according to the *T. reesei* genome version 2.0 database.
