# Supplementary figures and images for: Comparison of intracellular and secretion-based strategies for production of human α-galactosidase A in the filamentous fungus Trichoderma reesei
Source: BMC Biotechnol. 2014 Oct 27;14:91. doi: 10.1186/s12896-014-0091-y (PMC4219008; doi:10.1186/s12896-014-0091-y)

## Slide 1
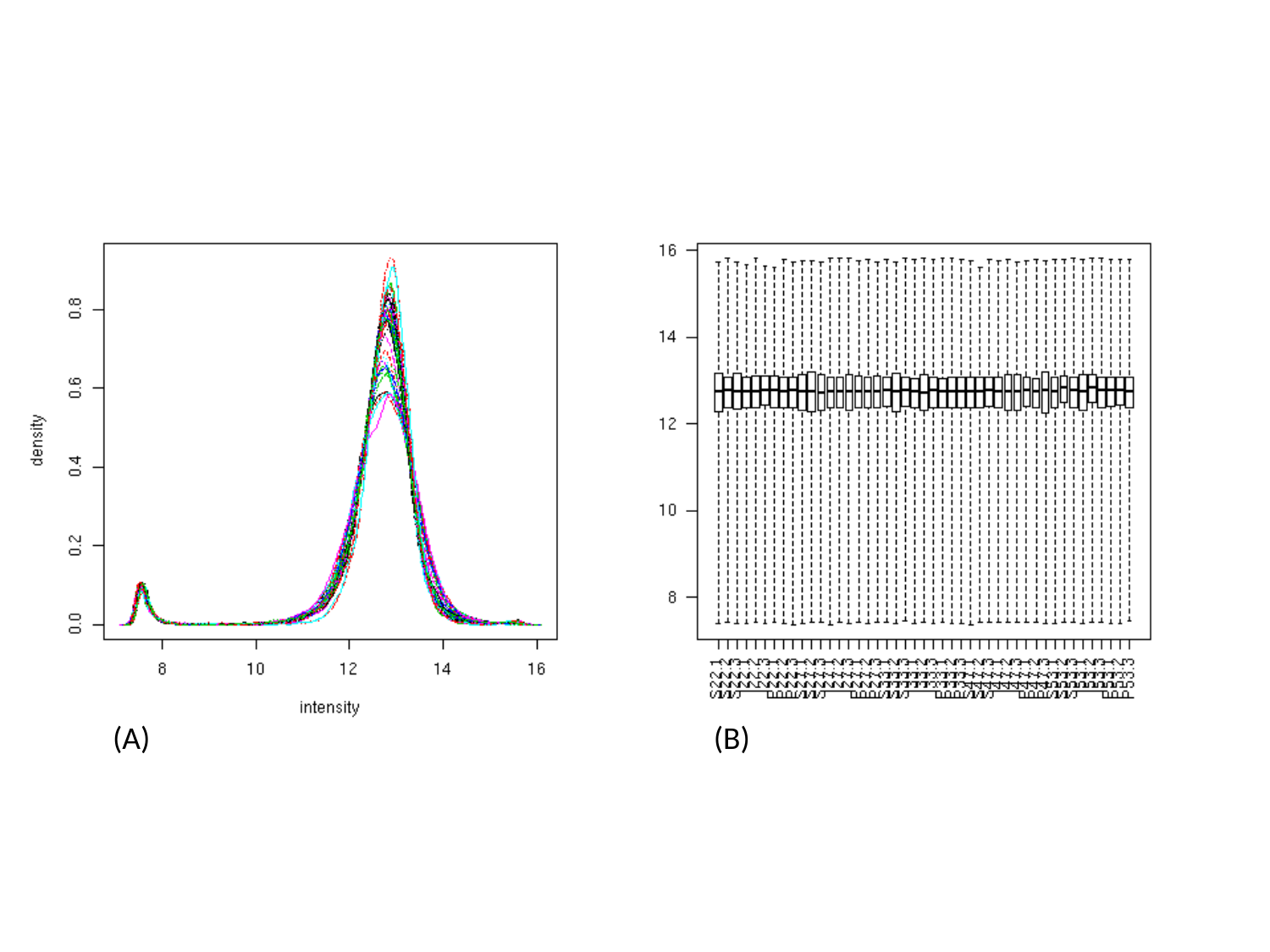

(A)
(B)

Supplement: Additional file 1: — Data quality of the microarrays. (A) Density histogram of rma normalized data. The variance of log2 intensities of the normalised arrays intensity fall within acceptable limits for comparison. (B) Boxplot of rma normalized data. The appropriate similarity of replicate samples was verified using principal component analysis in this two dimensional display. [file 12896_2014_91_MOESM1_ESM.pptx]
