# Supplementary material for: Comparison of intracellular and secretion-based strategies for production of human α-galactosidase A in the filamentous fungus Trichoderma reesei
Source: BMC Biotechnol. 2014 Oct 27;14:91. doi: 10.1186/s12896-014-0091-y (PMC4219008; doi:10.1186/s12896-014-0091-y)
Supplement: Additional file 2: — List of significant genes in all of the comparisons. Listing of all the significantly changing genes found above the fold change threshold in all transcriptional profiling comparisons according to the T. reesei genome version 2.0 database. [file 12896_2014_91_MOESM2_ESM.pptx]

## Slide 1
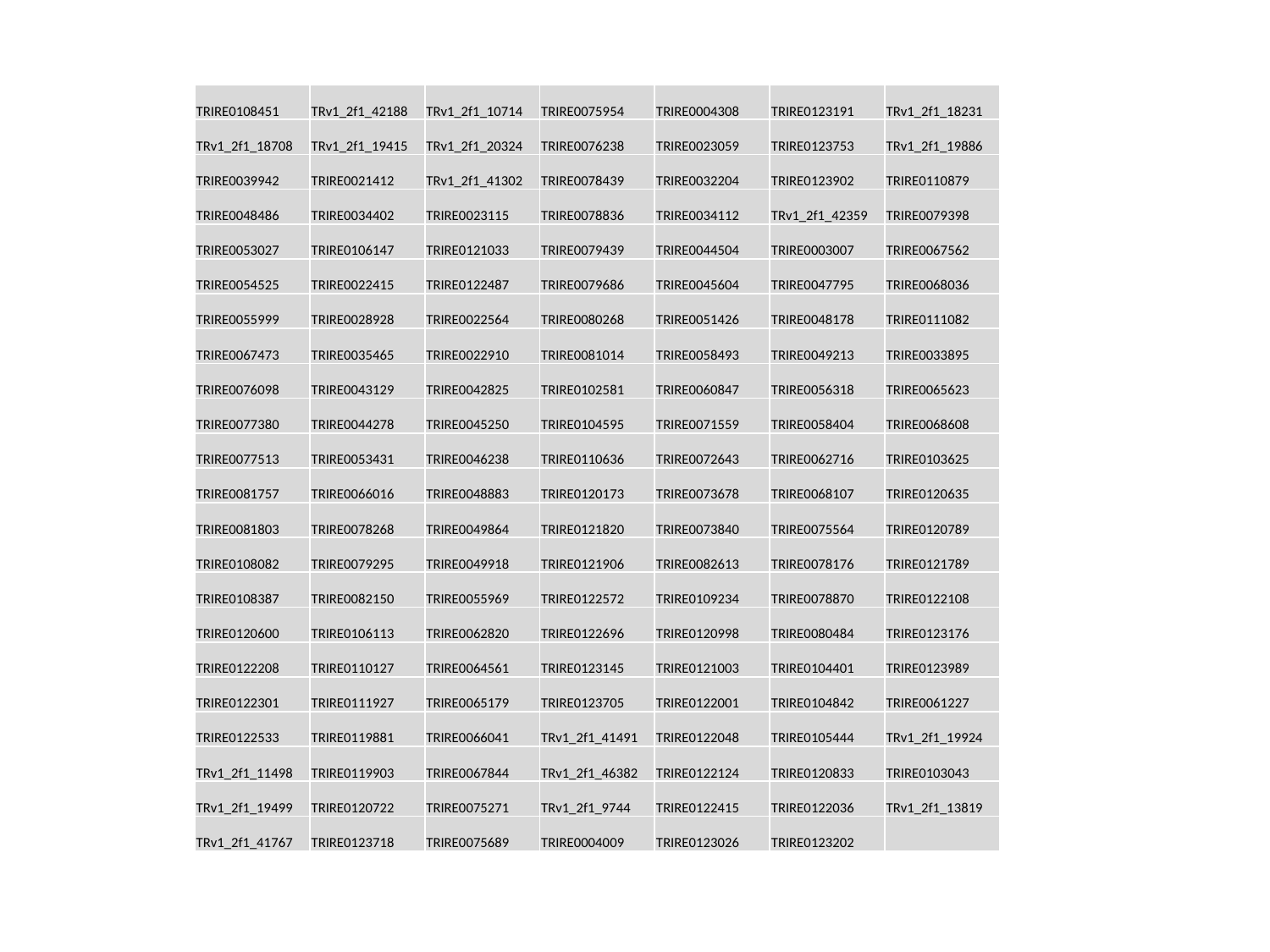

| TRIRE0108451 | TRv1\_2f1\_42188 | TRv1\_2f1\_10714 | TRIRE0075954 | TRIRE0004308 | TRIRE0123191 | TRv1\_2f1\_18231 |
| --- | --- | --- | --- | --- | --- | --- |
| TRv1\_2f1\_18708 | TRv1\_2f1\_19415 | TRv1\_2f1\_20324 | TRIRE0076238 | TRIRE0023059 | TRIRE0123753 | TRv1\_2f1\_19886 |
| TRIRE0039942 | TRIRE0021412 | TRv1\_2f1\_41302 | TRIRE0078439 | TRIRE0032204 | TRIRE0123902 | TRIRE0110879 |
| TRIRE0048486 | TRIRE0034402 | TRIRE0023115 | TRIRE0078836 | TRIRE0034112 | TRv1\_2f1\_42359 | TRIRE0079398 |
| TRIRE0053027 | TRIRE0106147 | TRIRE0121033 | TRIRE0079439 | TRIRE0044504 | TRIRE0003007 | TRIRE0067562 |
| TRIRE0054525 | TRIRE0022415 | TRIRE0122487 | TRIRE0079686 | TRIRE0045604 | TRIRE0047795 | TRIRE0068036 |
| TRIRE0055999 | TRIRE0028928 | TRIRE0022564 | TRIRE0080268 | TRIRE0051426 | TRIRE0048178 | TRIRE0111082 |
| TRIRE0067473 | TRIRE0035465 | TRIRE0022910 | TRIRE0081014 | TRIRE0058493 | TRIRE0049213 | TRIRE0033895 |
| TRIRE0076098 | TRIRE0043129 | TRIRE0042825 | TRIRE0102581 | TRIRE0060847 | TRIRE0056318 | TRIRE0065623 |
| TRIRE0077380 | TRIRE0044278 | TRIRE0045250 | TRIRE0104595 | TRIRE0071559 | TRIRE0058404 | TRIRE0068608 |
| TRIRE0077513 | TRIRE0053431 | TRIRE0046238 | TRIRE0110636 | TRIRE0072643 | TRIRE0062716 | TRIRE0103625 |
| TRIRE0081757 | TRIRE0066016 | TRIRE0048883 | TRIRE0120173 | TRIRE0073678 | TRIRE0068107 | TRIRE0120635 |
| TRIRE0081803 | TRIRE0078268 | TRIRE0049864 | TRIRE0121820 | TRIRE0073840 | TRIRE0075564 | TRIRE0120789 |
| TRIRE0108082 | TRIRE0079295 | TRIRE0049918 | TRIRE0121906 | TRIRE0082613 | TRIRE0078176 | TRIRE0121789 |
| TRIRE0108387 | TRIRE0082150 | TRIRE0055969 | TRIRE0122572 | TRIRE0109234 | TRIRE0078870 | TRIRE0122108 |
| TRIRE0120600 | TRIRE0106113 | TRIRE0062820 | TRIRE0122696 | TRIRE0120998 | TRIRE0080484 | TRIRE0123176 |
| TRIRE0122208 | TRIRE0110127 | TRIRE0064561 | TRIRE0123145 | TRIRE0121003 | TRIRE0104401 | TRIRE0123989 |
| TRIRE0122301 | TRIRE0111927 | TRIRE0065179 | TRIRE0123705 | TRIRE0122001 | TRIRE0104842 | TRIRE0061227 |
| TRIRE0122533 | TRIRE0119881 | TRIRE0066041 | TRv1\_2f1\_41491 | TRIRE0122048 | TRIRE0105444 | TRv1\_2f1\_19924 |
| TRv1\_2f1\_11498 | TRIRE0119903 | TRIRE0067844 | TRv1\_2f1\_46382 | TRIRE0122124 | TRIRE0120833 | TRIRE0103043 |
| TRv1\_2f1\_19499 | TRIRE0120722 | TRIRE0075271 | TRv1\_2f1\_9744 | TRIRE0122415 | TRIRE0122036 | TRv1\_2f1\_13819 |
| TRv1\_2f1\_41767 | TRIRE0123718 | TRIRE0075689 | TRIRE0004009 | TRIRE0123026 | TRIRE0123202 | |
